# Semi-solid Sucrose Stearate-Based Emulsions as Dermal Drug Delivery Systems

**DOI:** 10.3390/pharmaceutics3020275

**Published:** 2011-05-30

**Authors:** Victoria Klang, Julia C. Schwarz, Nadejda Matsko, Elham Rezvani, Nivine El-Hagin, Michael Wirth, Claudia Valenta

**Affiliations:** 1 Department of Pharmaceutical Technology and Biopharmaceutics, Faculty of Life Sciences, University of Vienna, Althanstraβe 14, 1090 Vienna, Austria; E-Mail: victoria.klang@univie.ac.at (V.K.); 2 Research Platform “Characterisation of Drug Delivery Systems on Skin and Investigation of Involved Mechanisms”, University of Vienna, 1090 Vienna, Austria;E-Mail: julia.schwarz@univie.ac.at; 3 Institute for Electron Microscopy and Fine Structure Research, Graz University of Technology and Centre for Electron Microscopy Graz, 8010 Graz, Austria; E-Mail: nadejda.matsko@felmi-zfe.at; 4 Department of Food Sciences and Technology, BOKU-University of Natural Resources and Applied Life Sciences Vienna, 1190 Vienna, Austria; E-Mail: elham.rezvani@boku.ac.at

**Keywords:** sucrose stearate S-970, O/W emulsion, semi-solid, nanoemulsion, particle size, skin penetration

## Abstract

Mild non-ionic sucrose ester surfactants can be employed to produce lipid-based drug delivery systems for dermal application. Moreover, sucrose esters of intermediate lipophilicity such as sucrose stearate S-970 possess a peculiar rheological behavior which can be employed to create highly viscous semi-solid formulations without any further additives. Interestingly, it was possible to develop both viscous macroemulsions and fluid nanoemulsions with the same chemical composition merely by slight alteration of the production process. Optical light microscopy and cryo transmission electron microscopy (TEM) revealed that the sucrose ester led to the formation of an astonishing hydrophilic network at a concentration of only 5% w/w in the macroemulsion system. A small number of more finely structured aggregates composed of surplus surfactant were likewise detected in the nanoemulsions. These discoveries offer interesting possibilities to adapt the low viscosity of fluid O/W nanoemulsions for a more convenient application. Moreover, a simple and rapid production method for skin-friendly creamy O/W emulsions with excellent visual long-term stability is presented. It could be shown by franz-cell diffusion studies and *in vitro* tape stripping that the microviscosity within the semi-solid formulations was apparently not influenced by their increased macroviscosity: the release of three model drugs was not impaired by the complex network-like internal structure of the macroemulsions. These results indicate that the developed semi-solid emulsions with advantageous application properties are highly suitable for the unhindered delivery of lipophilic drugs despite their comparatively large particle size and high viscosity.

## 1. Introduction

Sucrose fatty acid esters have been known as additives in the fields of nutrition, cosmetics and pharmacy for a long time [1-4]. However, it is only recently that their potential as mild non-ionic surfactants in the production of topical lipid-based drug delivery systems has been investigated more thoroughly. Different sucrose esters of various HLB values have been investigated for their potential to form O/W nanoemulsions [5-7] and to increase [8-10] or retard [11] the skin permeation of incorporated drugs. We have recently demonstrated the superiority of sucrose stearate S-970 over a comparable lecithin mixture to produce highly stable and homogeneous O/W nanoemulsions for dermal application [7]. In this context we found that the employed emulsifier sucrose stearate S-970 exhibited a peculiar rheological behavior when processed in a specific way. The astonishing gel-forming ability of sucrose stearate mixtures of intermediate hydrophilic/lipophilic balance (HLB value) around 9 to 11 has been reported in earlier literature [12,13] as well as recently [11,14,15]. However, no attempt has been made so far to design semi-solid multiphase vehicles which are stabilized in this fashion and can be used for incorporation of lipophilic drugs. The properties of a sucrose stearate/water dispersion were recently investigated [15], but no approach to incorporate additional excipients has been reported. However, incorporation of oil or model drugs may have a significant effect on the gelling properties of the sucrose ester and thus the microstructure of the resulting vehicles. Likewise, the drug release properties of such viscous sucrose ester-stabilized formulations have not been explored yet. Since these aspects are of great practical interest for the development of new formulations, the present work addresses these points.

A simple and skin-friendly sucrose stearate-based O/W emulsion was developed. Optical light microscopy, cryo transmission electron microscopy, rheological investigations and laser diffractometry were employed to characterize the microstructure of the novel system. Three lipophilic model drugs were incorporated and the effect of formulation microstructure and viscosity on the release profile of the system was investigated *in vitro* using franz-type diffusion cells and tape stripping experiments on porcine ears. Since a large particle size and high viscosity of a topically applied formulation are frequently associated with decreased or sustained drug release, we decided to compare the novel systems to corresponding O/W nanoemulsions. Through a slight alteration of the production process and subsequent high-pressure homogenization it was possible to develop fluid O/W nanoemulsions of the exact same composition as the novel macroemulsions and the same content of sucrose stearate, namely 5% (w/w). Microscopic and rheological investigations were performed on these systems as well. Dynamic light scattering and laser Doppler electrophoresis were employed to further characterize the nanoemulsions.

Overall, the aim of the present study was to explore the astonishing gel-forming properties of sucrose stearate S-970 in colloidal multiphase drug delivery systems and to characterize the nature of the resulting semi-solid vehicles. In addition, the drug release profile of these novel O/W emulsions was evaluated by comparing them to fluid nano-sized emulsions of identical chemical composition.

## 2. Experimental Section

### 2.1. Materials

Sucrose stearate (Ryoto Sugar Ester® S-970) was supplied by Mitsubishi-Kagaku Food Corporation (Tokyo, Japan). PCL-liquid (cetearyl ethylhexanoate, isopropyl myristate) was purchased from Dr.Temt Laboratories (Vienna, Austria). The preserving agent potassium sorbate was obtained from Herba Chemosan Apotheker-AG (Vienna, Austria). Flufenamic acid (CAS: 530-78-9, Batch No. 1619) and diclofenac acid (CAS: 15307-86-5, Batch No. 080304) were obtained from Kemprotec Limited (Middlesbrough, UK). Curcumin (CAS: 458-37-7, Batch No. 079K1756) was purchased from Sigma Aldrich (St. Louis, USA). Standard Corneofix® adhesive films with a square area of 4.0 cm^2^ were obtained from Courage + Khazaka GmbH (Cologne, Germany). All further chemicals used were of analytical reagent grade and used without further purification.

### 2.2. Formulations

Different concentrations of 1 to 5% w/w sucrose stearate S-970 were tested for their emulsifying potential in O/W emulsions. An increase in viscosity in dependence of the preparation method had been noticed especially for 5% w/w of sucrose stearate. At surfactant concentrations above 5% w/w, highly viscous creamy emulsions had been obtained irrespective of the preparation method, *i.e.* the emulsion microstructure had been too viscous to pass it through a high-pressure homogenizer even if heated. Therefore, an amount of 5% w/w of sucrose stearate was chosen for the final formulations.

The produced viscous macroscopic emulsions and the corresponding fluid nanoemulsions were identical in their composition, which is given in Table 1.

The preparation of a separate water and oil phase was the same in both cases. The aqueous phase, consisting of freshly distilled water and potassium sorbate, as well as the oil phase, consisting of the cosmetic oil PCL-liquid, were respectively stirred at 50 °C. Blank and drug-loaded formulations were prepared. The lipophilic model drugs flufenamic acid, diclofenac acid and curcumin were respectively dissolved in the oil phase at a concentration of 0.5% (w/w).

In case of the macroemulsions, sucrose stearate S-970 was dissolved in the oil phase. A highly viscous macroemulsion was obtained upon slow admixture of the aqueous phase, further stirring for 10 minutes and subsequent treatment with an ultra-turrax (Omni 500, 2500 rpm, 4 minutes). Refrigerated storage appeared to favor the observed gelling effect.

In case of the nanoemulsions, sucrose stearate S-970 was dissolved in the aqueous phase in order to maintain low viscosity of the system. The oil phase was subsequently added slowly to the aqueous phase for the same reason. The mixture was stirred for further 10 minutes, then pre-homogenized with an ultra-turrax (Omni 500, 2,500 rpm, 4 minutes). The mixture was stirred and heated to 50 °C before homogenization with a high-pressure homogeniser (EmulsiFlex C3, Avestin) for 16 homogenization cycles at 750 bars. Process parameters such as homogenization pressure and homogenization time had been optimized during previous studies [7]. Care was taken to process the mixture under mildly pre-heated conditions to avoid any sudden cooling and associated gelling within the device.

### 2.3. Emulsion Characterization

#### 2.3.1. Visual characterization

Both macroemulsions and nanoemulsions were investigated for the presence of structures visible to the eye as well as for their texture and skin feeling.

#### 2.3.2. Particle size

The macroemulsions were investigated for their particle size and particle size distribution by laser diffractometry (laser diffraction, static laser light scattering). Measurements were performed in triplicate on a Mastersizer 2000 (Malvern, UK). The instrument was operated with the HydroS 2000 sample dispersion unit (Malvern, UK) and software version 5.60 at 25 °C. The samples were diluted with distilled water (1:1000 v/v) and stirred slightly prior to analysis. All samples were analyzed in triplicate (n = 3). No sonification of the samples was performed prior or during the measurements to avoid the destruction of possible aggregates which may provide information about the microstructure as well as destabilization phenomena [16,17]. Laser diffraction measurements yield volume-weighted diameters as results for droplet sizes up to 2000 μm [16]. The parameters obtained in the present experiments were the d(v, 0.1), d(v, 0.5) and d(v, 0.9). Likewise, the volume weighted mean D [4,3] and the surface weighted mean D [3,2] were obtained. The d(v, 0.5) or volume median diameter marks the size where 50% of the distribution is above and 50% is below this value. The d(v, 0.1) represents the point where 10% of the volume distribution is below this value. In analogy, the d(v, 0.9) marks the point where 90% of the volume distribution is below this value. The d(v, 0.9) can be employed to quantify larger droplets which might be present in the sample such as aggregates formed due to internal processes or destabilization [16]. The volume weighted mean or volume mean diameter D [4,3] corresponds to the mean diameter of spheres with the same volume as the analyzed droplets and is calculated by d_4,3_ = ∑n_i_ d_i_^4^/∑ n_i_ d_i_^3^, where n_i_ is the number of particles with diameter d_i_ [17]. In analogy, the surface weighted mean D [3,2] represents the mean diameter of the spheres with the same surface area as the analyzed droplets. In addition, the span was automatically calculated for all samples. This dimensionless number represents the width of the droplet size distribution based on the 10%, 50% and 90% quantile. A wide particle size distribution will yield a comparatively large span. All parameters were calculated automatically using the Mie theory with a refractive index of 1.52 and a particle absorption value of 0.1. Measurements were conducted immediately after production and after six months of storage time.

The nanoemulsions were investigated for their particle size and particle size distribution by dynamic light scattering (DLS, photon correlation spectroscopy) using a Zetasizer Nano ZS (Malvern, UK) at 25 °C. Samples were diluted with freshly distilled water 1:100 (v/v) to diminish opalescence. The obtained parameters were the hydrodynamic diameter expressed as z-average value of the samples as well as the polydispersity index (PDI). The z-average value is an intensity weighted mean diameter of the bulk population of the sample while the PDI represents the particle size distribution within the formulations. PDI values below 0.2 indicate a narrow size distribution and thus good long-term stability [18]. The approximate measuring range of this instrument is from 3 nm to 3 μm [19]. All samples were analyzed in triplicate (n = 3). Each individual result was automatically calculated as the average of 3 measurements with 20 sub-measurements each. The particle size and PDI of the nanoemulsions were measured immediately after preparation and in regular intervals over a period of six months. In order to detect the presence of potential larger droplets or aggregates, both optical light microscopy and cryo TEM were employed.

#### 2.3.3. Particle surface charge (zeta potential)

The particle surface charge of the nanoemulsions was determined by laser Doppler electrophoresis using a Zetasizer Nano ZS (Malvern, UK). The electrophoretic mobility of the droplets was determined and automatically converted into the zeta potential using the Helmholtz-Smoluchowski equation by the Malvern data analysis software [16]. Zeta potential (ZP) values of the formulations were determined at 25 °C. Samples were diluted with distilled water (1:100 v/v) containing sodium chloride (0.01 mmol) in order to ensure constant conductivity below 0.05 ms/cm and thus reproducible measurement conditions [16,18,20]. The ZP roughly characterizes the surface charge of the emulsion droplets in solution. High absolute values above 30 mV lead to repulsive forces between the droplets which may improve the physical stability of multiphase systems [20]. Absolute ZP values above 60 mV indicate excellent electrochemical stability [16]. Zeta potential values were determined in triplicate for all nanoemulsions (n = 3) after production and in regular intervals over six months.

#### 2.3.4. Physical stability

The physical stability of both macroemulsions and nanoemulsions was compared by determination of the particle size after an observation period of six months. All formulations were stored at 4 °C. A visual control was regularly performed to detect any signs of instability. In case of the nanoemulsions, additional control measurements were performed in regular intervals over the whole observation period as well.

#### 2.3.5. Drug content, chemical stability and pH value

The drug content of all formulations was analyzed immediately after preparation to ensure appropriate drug incorporation. Briefly, 10 mg of the emulsion were dissolved in 1 mL of methanol, centrifuged for 6 minutes at 12,000 rpm (Hermle Z323K, MIDSCI, USA) and analyzed by HPLC or UV/Vis spectroscopy. Samples were taken at least in triplicate (n ≥ 3). In case of flufenamic acid and diclofenac acid, further samples were taken in regular intervals over three months to obtain a rough overview about the chemical stability of the acidic drugs and their homogeneous dispersion within the systems. Since curcumin is known to undergo a variety of degradation processes which would require more specific methods of analysis, no attempt was made to cover this aspect in the scope of the present work. The obtained data should merely ensure the use of representative and intact formulations for all further studies.

The pH value of the formulations was determined using a pH meter (Orion 420A, Bartelt, Austria). The pH of all formulations was determined at room temperature (25 °C) in regular intervals to detect destabilization phenomena induced by chemical degradation through hydrolysis or oxidation, which result in a decrease of pH [21].

#### 2.3.6. Optical light microscopy

Optical light microscopy was employed to obtain an overview about the presence of structures within the micrometer size range, which was especially interesting in the case of the macroemulsion morphology. In case of nanoemulsions, optical light microscopy served as a quality control to ensure the absence of larger structures. The microscopic analysis was carried out using a photo microscope (Zeiss Axio Observer.Z1 microscopy system, Carl Zeiss, Oberkochen, Germany) equipped with phase contrast and differential interference contrast (DIC) and using LD Plan-Neofluar objectives. A small amount of fresh or stored sample of the macroemulsions and nanoemulsions was respectively placed on an object slide, covered and analyzed immediately. Images were taken in conventional bright field mode, with phase contrast and with DIC while 5, 10, 20 and 40-fold magnifications were employed for all samples.

#### 2.3.7. Transmission electron microscopy (TEM): Cryo TEM and negative staining for conventional TEM

The structure of blank macroemulsions and nanoemulsions containing 5% (w/w) sucrose stearate was visualized using cryo TEM. To this end, several vitrified specimens were prepared for each formulation type. In case of the macroemulsion, a fresh glow-discharge copper grid covered with a perforated carbon film was placed on the sample, polished by filter paper in order to remove excess material and immediately plunged into liquid ethane. The macroemulsion samples were observed in an undiluted state so as not to destroy the internal structure. In case of the nanoemulsion, the sample was dissolved in distilled water (1:10 v/v). Subsequently, a 4 μm drop of the solution was placed on a fresh glow-discharge TEM copper grid covered with a perforated carbon film (Pelco International) and blotted with a filter paper to form a thin liquid film of the sample (thickness of 100–250 nm). The thinned sample was plunged into liquid ethane at its freezing temperature (−183 °C) to form a vitrified specimen, and was then transferred to liquid nitrogen (−196 °C) for storage until examination. Vitrified specimens were examined in a Philips T12 transmission electron microscope (Philips) operating at an accelerating voltage of 120 kV using an Oxford CT3500 (Oxford Instruments) cryo holder that maintained the vitrified specimens at −160 °C during sample observation. Images were recorded digitally on a cooled Gatan BioScan CCD camera (Gatan) using the DigitalMicrograph 3.4 software (Gatan) in low-dose imaging mode to minimize beam exposure and electron beam radiation damage.

In addition, conventional TEM analysis was performed for both samples after negative staining with uranyl acetate as previously described [22]. To this end, a carbon coated mesh was hydrophilized by glow discharge and a drop of the respective undiluted emulsion was deposited on the mesh. An aqueous solution of 1% (v/v) of the negative staining agent uranyl acetate was subsequently applied on the mesh to facilitate observation.

#### 2.3.8. Rheological characterization

The rheological properties of both macroemulsions and nanoemulsions were investigated. All experiments were performed on a Bohlin CVO Rheometer (Malvern Instruments, UK) with a thermostatic control system (Bohlin KTB30, Malvern, UK). The employed rheometer tools were a thermostatically controlled cone and plate tool with 40 mm in diameter and a 4° angle (CP 4°/40 mm) for the macroemulsions and a coaxial cylinder system with 25 mm in diameter (cup and bob C25) for the nanoemulsions. The temperature for both tools was maintained at 25 ± 0.5 °C throughout all experiments. The applied amount of sample was around 2 g for macroemulsions and around 15 g for nanoemulsions. Both blank and drug-loaded formulations were investigated. All measurements were performed at least in triplicate (n ≥ 3). Values given in figures and tables are the average values.

Flow curves were established for all systems. The flow properties of both blank and drug-loaded macroemulsions and nanoemulsions were investigated by measuring the dynamic viscosity η (in Pa·s) under shear stress. Rheological experiments in a controlled-rate mode were performed. A controlled shear rate *γ* was employed at a constant temperature of 25 ± 0.5 °C to determine the viscosity of the samples as a function of the shear rate ranging from 0.1 s^−1^ to 100 s^−1^. The obtained flow curves of both macroemulsions and nanoemulsions were compared to the power law model (Ostwald model) defined as *η* = *m γ* ^(*n*−1)^ where *η* represents the shear viscosity, *m* equals the consistency coefficient, *γ* represents the shear rate and *n* is the flow behavior index. Values of n = 1 are indicative for Newtonian fluids while values of n < 1 are representative for shear-thinning, *i.e.* pseudoplastic fluids. Values of n > 1 are found for shear-thickening dilatant fluids [23,24].

Moreover, oscillatory shear experiments were performed for all systems. In the oscillatory mode, a sinusoidal stress is applied to the sample and the response in form of the induced strain is measured [25,26]. To this end, the rheometer tool is moved forward and backward in a defined sinusoidal motion. After identification of the linear viscoelastic region the samples were investigated over a frequency of 1–10 Hz. Crucial parameters were determined as a function of the oscillatory frequency (*v*, in Hz). The parameters obtained in this fashion are the elastic modulus G′, the viscous modulus G″, the complex modulus G* and the dynamic viscosity *η*′.

The elastic modulus or storage modulus G′ is defined as G′ = G* cos (δ) and describes the recoverable energy that is stored within an elastic system. The viscous modulus or loss modulus G″ is calculated by G″ = G* sin (δ) and represents the energy that is dissipated in the viscous flow and transformed into heat. These moduli represent the real and imaginary parts of the complex dynamic shear modulus G* [25–27]. The dynamic viscosity *η*′ is defined as *η*′ = G″/ω, where ω is the angular velocity of the oscillatory stress which is in turn related to the oscillatory frequency by the relationship ω=2*πv*. The related phase angle is expressed as δ [25,26].

### 2.4. Skin permeation experiments using franz-type diffusion cells

*In vitro* skin permeation studies were performed using standard franz-type diffusion cells (Permegear, USA). Thus, the potential influence of the different formulation properties of macroemulsions and nanoemulsions on the skin permeation of the three model drugs flufenamic acid, diclofenac acid and curcumin was investigated. Porcine abdominal skin was chosen as model membrane because of its morphology and permeability, which are similar to those of human skin [9,28]. The porcine abdominal skin was freed from hair and treated with a dermatome (GB 228R, Aesculap) set at 1.2 mm. The skin was stored at −20 °C until use and thawed prior to the experiments. Appropriate skin patches were clamped between the donor and the receptor chamber of the diffusion cells having a permeation area of 1.13 cm^2^. The receptor compartment was filled with 2 mL of phosphate buffer (pH 7.4, 0.012M) in case of flufenamic acid and diclofenac acid [29]. In case of curcumin, a mixture of distilled water/ethanol (50/50 % v/v) was employed to ensure sink conditions [30,31]. The diffusion cells were kept at skin surface temperature (32 °C) and stirred with magnetic bars for 24 hours. The formulation (0.6 g) was placed on the excised skin in the donor chamber. Samples of 200 μL were removed at defined time intervals for analysis and were replaced by fresh receptor medium. At least five parallel experiments were performed for each formulation (n > 5). The samples were analyzed for their drug content by HPLC in case of flufenamic acid and diclofenac acid or by UV/Vis spectroscopy in case of curcumin. Permeation profiles of the drugs were constructed by plotting time (hours) against the cumulative amount of the drug (μg/cm^2^) as measured in the receptor solution. In addition, the steady state flux (J, μg·cm^−2^·h^−1^) was calculated by linear regression after the respective lag-times.

### 2.5. Skin penetration experiments via in vitro tape stripping

In order to obtain penetration profiles of the three model drugs from both macroemulsions and corresponding nanoemulsions at least six individual tape-stripping experiments were performed for each formulation (n ≥ 6) on porcine ear skin. Fresh porcine ears were donated by the Clinic for Swine, University of Veterinary Medicine, Vienna. Since previous studies had shown that frozen storage does not alter the skin barrier function or the stratum corneum properties in the context of tape stripping experiments [32,33], the ears were stored at −24 °C and thawed prior to the respective experiments. After defrosting, the ears were cleaned carefully with purified water and blotted dry with soft tissue. The skin was subsequently freed from hair with scissors and intact, representative skin areas were indicated with a permanent marker. In addition, the transepidermal water loss (TEWL) of the skin was determined using the closed-chamber device AquaFlux® (Biox Ltd., London, UK) to confirm an intact skin barrier function and to monitor the defrosting process [33]. The software program AquaFlux® V6.2 was used for analysis of these data. When the TEWL reached values between 15 and 20 g m^−2^ h^−1^ the experiment was started. To this end, the porcine ears were stretched out on styrofoam plates and fixed with needles if necessary. The respective formulation was applied onto the marked skin area at a concentration of 6 mg·cm^−2^ with a saturated vinyl glove finger and was gently distributed and massaged for 30 seconds. After a penetration time of 1 hour the tape stripping procedure was started.

The adhesive films employed to remove the superficial stratum corneum layers were standard Corneofix® tapes. Care was taken to ensure a reproducible working procedure. After placing the first adhesive film on the skin its outline was indicated with a permanent marker to ensure subsequent tape stripping on the exact same location. Pressure was applied with the thumb covered in a vinyl glove as previously reported [34] to ensure a rolling movement and thus minimize the influence of wrinkles [35]. The experiment was performed on a balance to ensure a constant pressure of 49 N (5 kg) which is a prerequisite for the removal of reproducible amounts of stratum corneum proteins [33]. After applying pressure for 3 seconds, the tape was removed in a single rapid movement. In this fashion, 20 sequential adhesive films were removed per experiment. The amount of adherent corneocytes was subsequently determined by IR-densitometry using the infrared densitometer SquameScan™ 850A (Heiland electronic GmbH, Wetzlar, Germany) [36]. Briefly, the optical pseudo-absorption of the adhesive films at a wavelength of 850 nm is employed to quantify the amount of stratum corneum proteins on the tapes. The absorption values in % can be read from the display and the corresponding mass of proteins can be determined using the correlation factor of 0.41 which was previously established for analysis of porcine ear skin [33]. This value can be employed to calculate the mass of SC proteins (*m*) after determination of their pseudo-absorption at 850 nm (*A*) for a normalized tape area of 1 cm^2^ by employing the equation m = A/0.41 (in μg·cm^−2^).

The mean cumulative amount of removed stratum corneum proteins was employed to establish the penetration depth of the applied drugs in relation to the complete stratum corneum thickness. The latter was determined by continuous stripping of the complete stratum corneum in four of the experiments (n = 4) until the detection limit of the IR-densitometer was reached.

### 2.6. HPLC analysis

The formulations or samples containing flufenamic acid or diclofenac acid were analyzed for their drug content by HPLC (Series ISS-200, Perkin Elmer, USA), consisting of an auto sampler, a lc pump and an UV-diode array detector (235C). Previously reported methods were used using a Nucleosil 100-5 C18 column (250 mm × 4 mm, Macherey-Nagel, USA) plus a Nucleosil 100-5 C18 pre-column (CC8/4, 40 mm × 4 mm, Macherey-Nagel, USA). For all analyses, the oven temperature was set at 50 °C and the injection volume was 20 μL. The analysis of the data was performed using the TotalChrom Navigator 6.2.0 software. Standard solutions of the drugs were prepared and calibration curves were calculated by plotting the analyzed drug concentrations against the obtained peak area values.

The quantification of flufenamic acid and diclofenac acid was conducted according to previously described methods [29,37]. For both drugs, the mobile phase consisted of methanol/water (75/25 w/w); glacial acetic acid was added until a pH value of 3.2 was reached. The flow rate was 1.0 mL/min in both cases. For flufenamic acid, the detection wavelength was set at 245 nm with a retention time of 4.5 minutes. A calibration curve was calculated based on peak area measurements of diluted standard solutions ranging from 0.09 μg/mL to 110.50 μg/mL with a coefficient of determination R^2^ = 0.9999. The limit of detection for flufenamic acid was found to be around 0.04 μg/mL; the limit of reasonable quantification was set at 0.09 μg/mL. For diclofenac acid, the detection wavelength was set at 280 nm with a retention time of 10 minutes. The measurement range of the diluted standard solutions employed for the calibration curve was between 0.06 μg/mL and 28.97 μg/mL with R^2^ = 1. The limit of detection for diclofenac acid was, likewise, around 0.04 μg/mL; the limit of quantification was set at 0.06 μg/mL.

### 2.7. UV/VIS spectroscopic analysis

The quantification of curcumin was performed as previously reported [30] using a double beam UV/Vis spectrophotometer (Spectrophotometer U-3010, Hitachi, Japan). A correlation curve was established for standard solutions of curcumin in pure ethanol (96% v/v) ranging from 0.31 μg/mL to 5.01 μg/mL with R^2^ = 0.9952. The curcumin content of the samples filled into quartz cuvettes was determined at 425 nm. Samples containing a higher curcumin content were diluted prior to the analysis until values within the linear range of the calibration curve were obtained. This was frequently the case for samples extracted from the first two adhesive films of the tape stripping experiments.

### 2.8. Statistical data analysis

Results are expressed as means of at least three experiments ± SD. Statistical data analyses were performed with the software program GraphPadPrism3. Parametric data were analyzed using the Student's t-test with P < 0.05 as minimum level of significance while non-parametric data were analyzed using the Mann-Whitney test or the Wilcoxon signed rank test with P < 0.05, respectively.

## 3. Results and Discussion

### 3.1. Formulations and production aspects

The optimal surfactant concentration to produce both highly viscous macroemulsions and fluid nanoemulsions was found to be 5% w/w. Despite the increasing viscosity, considerate sample preparation still allowed for high-pressure homogenization of the mixture in case of the anoemulsions. Thus, the physicochemical properties of emulsions stabilized by 5% w/w of sucrose stearate could be varied by modification of the production process.

As anticipated, conductivity measurements confirmed the nature of the obtained emulsions as being of the O/W type. The temperature seemed to play a crucial role during processing of the sucrose stearate-based emulsions. If the fluid mixture was kept at 40–50 °C, further homogenization was feasible. If the mixture was cooled, some sort of gelling effect took hold and transformed the system into a semi-solid emulsion. This was especially pronounced if the mixture was cooled down rapidly. Similar behavior has been reported for aqueous sucrose stearate dispersions [15]. Moreover, the different ways of emulsion preparation as described in the literature [22] may have had an additional influence on the properties of the resulting mixture and thus either allowed for further high-energy processing or not. The variation in the mixing protocol of the compounds apparently influenced the viscosity of the pre-emulsion, which plays an important role for later processing [19].

### 3.2. Visual characterization

Creamy, thick macroemulsions were produced by dissolving the sucrose ester in the oil phase. The low density of the emulsions suggested that large amounts of air were incorporated to provide a fluffy appearance. All formulations had an appealing, homogeneous appearance which was retained over the whole observation period.

**Figure 1.**
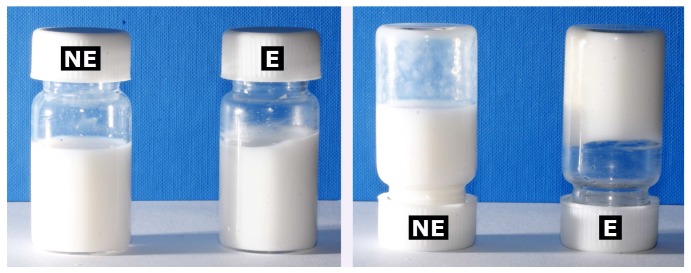
Visual appearance of a sucrose stearate-based macroemulsion (E) and a corresponding nanoemulsion (NE). Age of the presented formulations: 9 months.

In contrast, fluid if slightly gel-like nanoemulsions were produced by dissolving the surfactant in the aqueous phase and subsequent high-pressure homogenization. Figure 1 shows the visual appearance of the viscous macroscopic emulsions in contrast to the fluid nanoemulsions. Upon closer inspection, small white aggregates could be detected in the otherwise homogeneous whitish to bluish nanoemulsions. These were most likely composed of surfactant aggregates formed during or after high-pressure homogenization and can be ascribed to the comparatively high surfactant concentration as opposed to our previous studies [7]. These structures did however not impair the performance or physical stability of the formulations in any way. Upon cooled storage at 4 °C, they appeared to contribute to the increasingly gel-like texture of the nanoemulsions. Indeed, low storages temperatures have previously been reported to cause gelation of nanoemulsions of certain compositions [38].

### 3.3. Particle size

In case of the macroemulsions, the mean particle sizes were in the micrometer range as shown in Table 2A and 2B.

All parameters of importance are given, namely the D [4,3], D [3,2], d(v, 0.1), d(v, 0.5) and d(v, 0.9) values as well as the span. The span, which describes the width of the particle size distribution, was comparatively large for both blank and drug-loaded formulations. This indicates that various emulsion droplet sizes, ranging from the nanometer up to the micrometer scale, were present in the mixture. This could also be derived from a visual observation of the obtained distribution curves, which indicated a highly polydisperse nature of the samples with droplet sizes ranging from 0.7 up to several hundred micrometers. This was especially pronounced after 6 months of storage. In particular the strong increase in d(v, 0.9) values points to a small population of increasingly large individual droplets.

Since the samples were diluted prior the measurements, it is unclear whether these data merely give an overview about the oil droplet size distribution within the emulsion, or also about remnants of the hydrophilic network which provides the basis for the high viscosity of the systems. Likewise, since the results obtained with this technique are volume-based, the presence of a few individual larger droplets may skew the correlation considerably since the presence of very small droplets may easily be outweighed by the far more voluminous larger droplets. Thus, an additional microscopic analysis was performed.

In case of the nanoemulsions, the mean particle sizes as determined by DLS were in the lower submicron range for both blank and drug-loaded formulations (Table 3).

The narrow intensity-based distribution curves indicated a monodisperse nature of the samples. The PDI values were below 0.2 except for curcumin-loaded formulations, which indicates a narrow droplet size distribution and thus good long-term stability. Individual whitish aggregates detected among visual inspection were not detected with these measurements since they were most likely dissolved upon dilution of the samples. It is likewise known that DLS alone may fail to detect the presence of individual larger oil droplets or vesicles and may thus provide incomplete information [39]. Therefore, as for the macroemulsions, an emphasis was placed on microscopic examination techniques to obtain more accurate information about the actual microstructure of the formulations.

In context with the DLS measurements it has to be mentioned that the highly lipophilic drug curcumin was rather dispersed than dissolved within the oil phase. The presence of undissolved drug did not disturb the particle size measurements, as previously reported [40]. Merely a slight increase of the PDI value was noticed. Homogeneous distribution of the drug could be obtained after re-homogenization of the system by slight shaking. In case of the corresponding macroemulsions with curcumin the drug was homogeneously distributed within the system and no dispersed drug aggregates were visible within the homogeneous orange emulsion.

Overall, the droplet size in equilibrated emulsions is mainly determined by the intensity of mechanical agitation, the amount and interfacial properties of the surfactants, the interfacial tension and the physical properties of the oil and aqueous phases [17,41]. The properties of an emulsion will change significantly with the type of emulsification process used [41], as was impressively demonstrated in the present study. Thus, care should be taken to ensure an exact mixing procedure, since any irregularity may lead to changes in formulation microstructure.

### 3.4. Particle surface charge of nanoemulsions

The ZP values of blank and drug-loaded nanoemulsions are given in Table 3. The surface charge of sucrose stearate-based nanoemulsions was in the range around −50 to −60 mV, which indicates high electrochemical stabilization of the system. These high negative values are most likely caused by the presence of residual free fatty acids at the interface [7].

### 3.5. Physical stability

**Figure 2.**
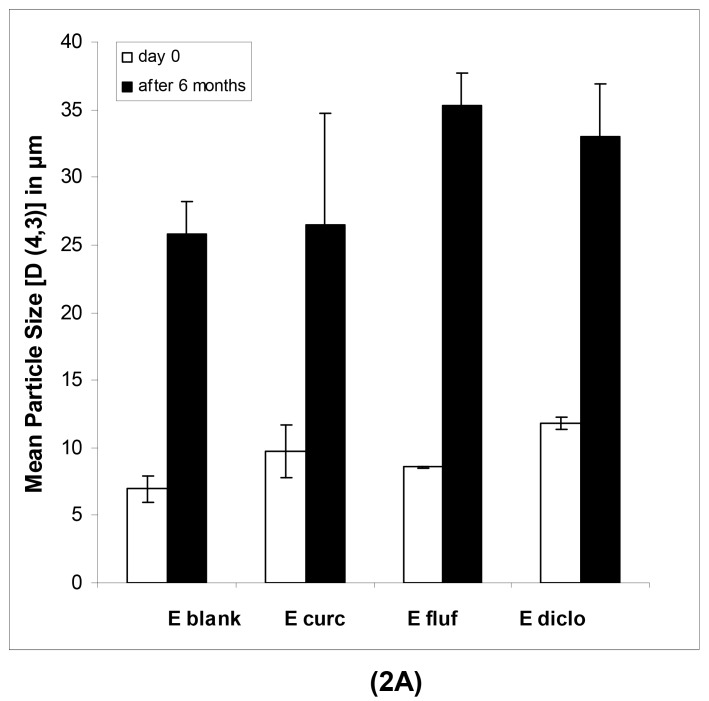

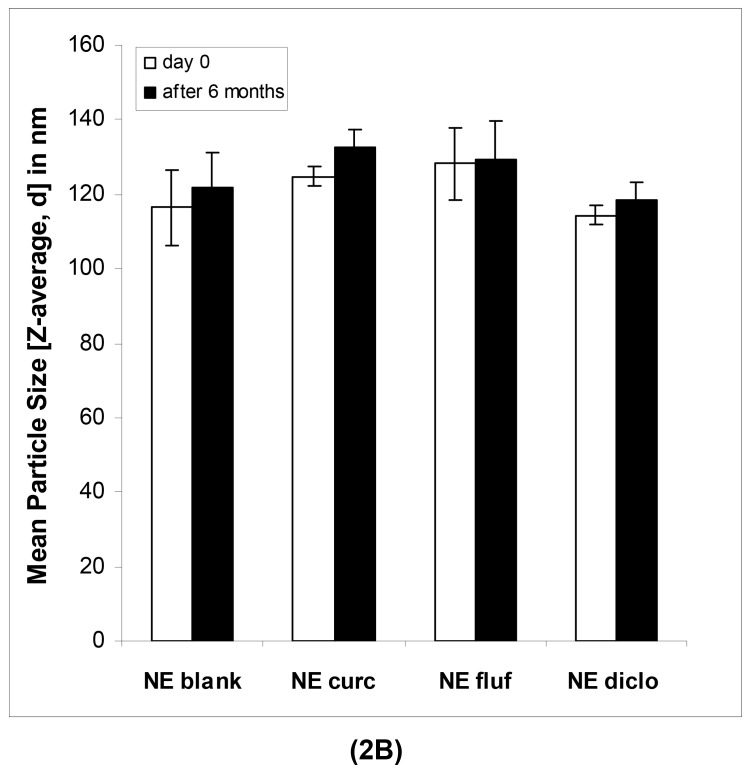
Mean droplet size of blank and drug-loaded macroemulsions (E, **2A**) and corresponding nanoemulsions (NE, **2B**) as determined immediately after production and after 6 months. The diluted samples were analyzed in triplicate at 25 °C on a Mastersizer 2000 (Malvern, UK) in case of the macroemulsions and a Zetasizer Nano ZS (Malvern, UK) in case of the nanoemulsions. The parameters shown are the volume weighted mean D [4,3] for macroemulsions and the mean droplet diameter as z-average for nanoemulsions. Values represent the mean of at least three formulations (n ≥ 3) and are given ± SD.

The physical long-term stability of all systems was highly satisfying. As anticipated in case of the macroemulsions, a notable increase in mean particle size after six months was observed (Figure 2A). However, the formulation remained visually stable and showed no signs of phase separation or microbial contamination upon conventional storage at 4 °C in a receptacle for ointments.

In case of the nanoemulsions (Figure 2B), the mean particle sizes remained highly constant in the course of six months as confirmed by regular measurements.

As can be seen in Table 4, this was the case for both blank and drug-loaded nanoemulsions, shown on representative formulations containing flufenamic acid. The mean ZP values of the nanoemulsions increased slightly (Table 4A and 4B), most likely due to chemical changes within the formulations such as hydrolysis of surfactant molecules or excipients of the oil phase. This process results in the release of free fatty acids and thus an increased amount of negative charges at the interface. Since a certain surplus of surfactant was employed, the chemical degradation of the latter may have been more apparent than in our previous studies [7]. At the same time, this surplus of surfactant might be responsible for the individual whitish aggregates that were observed within the nanoemulsions. The number of these aggregates visible to the eye increased over time.

In summary, particle size measurements of submicron-sized emulsions by light scattering techniques are not always sufficient to monitor the actual physical stability of such formulations. Visible destabilization phenomena such as separation of oil droplets or formation of a precipitate might remain undetected [40]. Thus, carefully visual inspection of the samples is necessary. Additional and more precise information can be gained by microscopic investigations.

### 3.6. Drug content, chemical stability and pH value

The initially determined drug contents of all formulations as well as the chemical stability of flufenamic acid and diclofenac acid remained satisfying over the course of a limited observation period of three months. No further attention was devoted to this topic since a more thorough analysis of the chemical stability of the incorporated drugs, especially in case of curcumin, was not an emphasis of this study.

The pH values of blank formulations were around 6.80 ± 0.04 for macroemulsions and 6.89 ± 0.01 for nanoemulsions (n = 3, respectively). Drug incorporation led to slightly lower pH values in case of flufenamic acid and diclofenac acid (data not shown). However, all pH values were in an acceptable range for dermal application.

Interestingly, chemical degradation processes appeared to affect the nanoemulsions to a greater extent than the corresponding macroemulsions irrespective of drug incorporation. After a storage time of 6 months, the overall mean pH value of all formulations was 6.40 ± 0.20 in case of the macroemulsions and 6.01 ± 0.11 in case of the nanoemulsions (n = 13, respectively). This observation is in agreement with previously reported data which indicate that lipid oxidation in nanoemulsions proceeds more rapidly than in conventional emulsions due to the increased surface area of the nano-sized droplets [17].

### 3.7. Optical light microscopy

Optical light microscopy was conducted to obtain an overview about larger structures present in the different formulations. The images were in good agreement with the visual observations of the samples as well as the particle size measurements.

**Figure 3.**
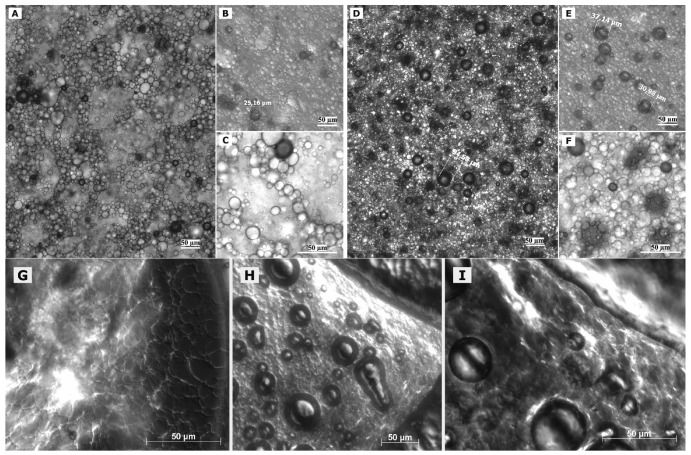
Images of fresh as well as stored macroemulsions as observed by optical light microscopy. Images **3A, 3B and 3C** show a blank macroemulsion directly after preparation while the corresponding images **3D, 3E and 3F** show the same formulation after 6 months of storage. The employed microscopic modes were 10× magnification/bright field (3A, 3D), 20× magnification/DIC (3B, 3E and 3G, 3H, 3I) and 40× magnification/bright field (3C, 3F). The indicated scale bars represent 50 μm. Images **3G, 3H and 3I** represent artefacts of fresh macroemulsions obtained after 20 minutes of storage of the object slide.z

In case of freshly prepared blank macroemulsions a complex network of hydrophilic spherical structures presumably composed of sucrose ester/water aggregates was observed (Figure 3A to 3C). Large spherical interconnected aggregates formed a net-like structure which surrounded the darker oil droplets. In between the large “meshes” of this network, smaller spherical aqueous structures were observed which filled up the space. Likewise, it may be assumed that large amounts of air are incorporated as well. Overall, the particle sizes observed for the different surfactant aggregates varied from around 30 μm for the large network-forming structures down to 0.6 μm for the smaller aggregates filling up the spaces in between. The oil droplets were of variable size and their diameter ranged mostly within the micrometer range, as already indicated by the laser diffraction measurements. Although these particle size measurements were conducted with highly diluted samples, it is unclear whether the sucrose ester aggregates were included in the analysis. If this was the case, it would account for the high polydispersity of the analyzed samples. Particle sizes as small as 600 nm have likewise been reported for the structural compounds of aqueous gel-like dispersions of sucrose stearate [15]. However, the values reported by Ullrich and co-workers were based on DLS analysis of highly polydisperse and undiluted samples, which renders an interpretation difficult.

Interestingly, the nature of the viscous macroemulsions changed upon storage. Images that were taken after a storage time of over 6 months revealed an increasingly dense aggregate network with less free space between the aggregate “meshes” of the network (Figure 3D to 3F). This increase in density of the network might have been caused by a minor separation of water condensation on the lid of the storage receptacle. Likewise, the influence of gravity might have caused incorporated air to escape from the emulsion bulk phase. As anticipated by particle size measurements, the average size of the dispersed oil droplets showed a definite increase with oil droplet diameters frequently larger than 30 μm. Individual oil droplets of up to 160 μm in diameter were observed as well. It may be assumed that the presence of such individual large droplets remained undetected in the laser diffraction measurements due to methodological limitations [42]. Despite this obvious change in the internal formulation structure, the visual appearance of the semi-solid vehicles remained intact.

Interesting additional information was gained from observation of changes in formulation microstructure upon manipulation on an object plate. After prolonged presence of the macroemulsions on the object plate, a dried network became visible which covered the dried out area around residual water droplets (Figure 3G). In addition, the potential of the sucrose ester mixture to form liquid crystalline structures became more apparent as increasingly large areas, also along the dried network structure, showed birefringence under polarized light (Figure 3G, 3H, 3I). These observations indicate that the network structure of the viscous formulation might be at the margin of a weak liquid crystalline matrix.

Overall, the observed network of unilamellar vesicles bears a certain resemblance to a cubic gel phase observed in previous studies [43,44]. Despite the apparent differences in formulation composition and size range when compared to our macroemulsions, similar general observations were reported. A system of high viscosity was obtained with no other compounds than a dispersed phase fraction of 5–15% w/w. The vesicular unilamellar spheres responsible for the system's properties consisted of amphiphilic material only. Dilution of the gel phase led to the formation of a viscous, but not gel-like phase which was unfortunately not further characterized. The fact that the macroemulsion structure is not as ordered as the cubic gel structure might be due to the fact that much larger amounts of water and less surfactant are present. Our system might thus be comparable to the described diluted cubic phase system. Another explanation might point to a resemblance to self-standing gel-like emulsions. However, larger amounts of internal phase are usually reported for such systems [45].

**Figure 4.**
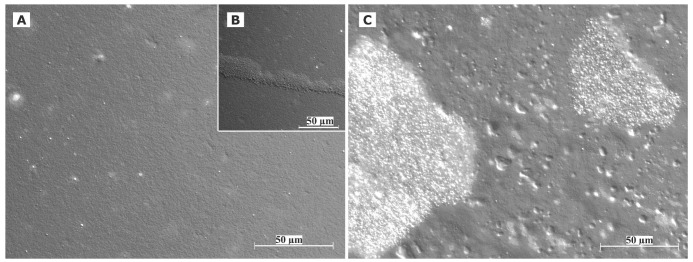
Images of fresh, as well as stored, nanoemulsions as observed by optical light microscopy. All images were taken with 40 × magnification/DIC. Image **4A** shows the blank nanoemulsion directly after preparation. Figure **4C** shows the same formulation after 6 months of storage. Image **4B** shows an artefact obtained with a fresh nanoemulsion sample due to evaporation effects on the object slide. The indicated scale bars represent 50 μm.

In case of the freshly prepared nanoemulsion (Figure 4A), constant background movement indicated the presence of nano-sized oil droplets subjected to Brownian motion [22]. The samples were highly homogeneous except for a few larger vesicular structures and individual surfactant aggregates. These structures which were visible to the naked eye as whitish flakes appeared as irregularly shaped “islands” within the formulation. However, their number was highly limited after production. These aggregates remained undetected during the DLS measurements of the diluted samples.

The nature of the fluid nanoemulsions changed upon storage. Images that were taken of samples after 6 months of storage time (Figure 4C) revealed a much larger number of the described shapeless aggregates. It may be assumed that these aggregates represent separated fractions of surplus sucrose ester. For blank nanoemulsions, the viscosity of the sample appeared to be increased as compared to the fresh one due to gelling effects of the surfactant. No such gelling effect upon storage was observed for the more fluid nanoemulsions with flufenamic acid and diclofenac acid. This aspect is currently being investigated in rheological studies over prolonged observation periods and will be reported in a separate context. Overall, nanoemulsions apparently suffered notable changes in formulation microstructure despite the fact that the particle size measurements suggested otherwise.

Again, observation of the sample's behavior on the object plate provided useful information. The nanoemulsion droplet started gelling immediately upon application on the plate. After covering of the sample, a structured dispersed film remained at the edges of the droplet (Figure 4B). Again, this rather ordered structural network may be situated at the margin of a weak liquid crystalline network [15]. Similar behavior can be observed upon positioning of a microemulsion on an object plate, where growth of liquid crystals can be observed at the margin of the sample due to evaporation of volatile compounds.

Although the peculiar rheological behavior of colloidal sucrose ester suspensions in water has recently attracted attention [11,14], the microstructure of these systems is still somewhat unclear [15]. Microscopic data exists mostly for suspensions with higher concentrations of sugar surfactant, which consequently contain increasing amount of liquid crystalline structures [13]. Interestingly, for a suspension of another sucrose stearate mixture with a slightly higher HLB value of 12, liquid crystalline structures were already observed at a concentration of 4% w/w [46]. Although information on the structure of O/W emulsions co-stabilized by alkylpolyglucosides, a frequently employed type of sugar surfactant, can be found [47], no such data exist for O/W emulsions stabilized by a sucrose ester mixture alone.

### 3.8. Cryo TEM and TEM after negative staining

**Figure 5.**
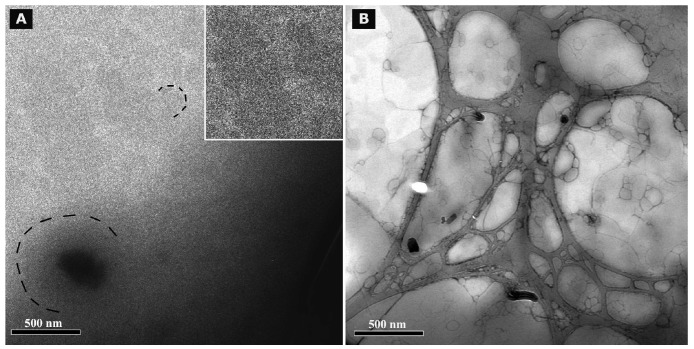
Analysis of the microstructure of blank macroemulsions by cryo TEM **(5A)** and conventional TEM after negative staining with uranyl acetate **(5B)**. The magnification is illustrated by the black scale bars.

Cryo TEM investigations of freshly prepared formulations were additionally performed to visualize the structural differences between the two original systems on a nano-scale level. In case of the macroemulsion (Figure 5A), a dense structural network was observed which appeared to consist of closely located or connected spherical aggregates with mean diameters as small as a few hundred nanometers (marked areas). These aggregates formed the larger droplets which had been visualised by light microscopy. Since the sample was observed in its original state without dilution and the TEM image is merely a 2D projection, the borders of the smaller aggregates could not be clearly distinguished. However, the images serve to confirm the rich internal structure of the macroemulsion. The structural network appeared to be remarkably homogeneous, since the ice crystals commonly observed during cryo-preparation emerged in a highly ordered and regular fashion. Additional analysis with conventional TEM sample preparation and negative staining confirmed these observations (Figure 5B). Droplets of various sizes could be distinguished.

**Figure 6.**
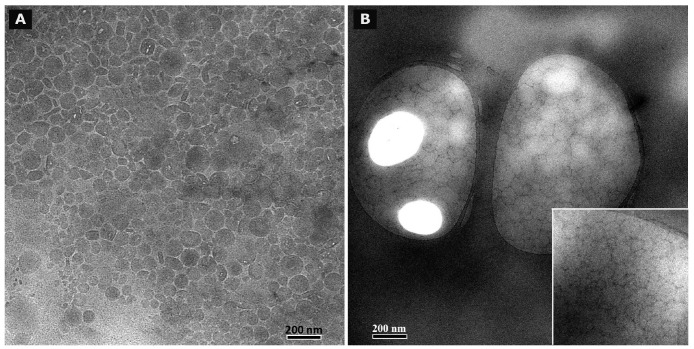
Analysis of the microstructure of blank nanoemulsions by cryo TEM **(6A)** and conventional TEM after negative staining with uranyl acetate **(6B)**. The magnification is illustrated by the black scale bars.

In case of the nanoemulsion, a common if highly crowded internal structure was revealed despite the dilution of the sample (Figure 6A). The nano-sized oil droplets appeared to be partially deformed or collapsed, which is not entirely unexpected given the high surfactant concentration and resulting high viscosity of the formulation. Likewise, the shape of the deformable nanodroplets can be influenced by the oil volume fraction [48]. At high oil volume fractions, the surfaces of the crowded droplets strongly repel each other. This can cause the droplets to deform and become non-spherical [49]. In addition, individual shapeless aggregates most likely composed of sucrose stearate/water were detected in the nanoemulsion sample, which confirmed the optical light microscopy data. The rheological properties of the sample influence the processing conditions during high-pressure homogenization. The equilibrium between the rupturing of oil droplets and coalescence events which occur within the device are governed by the viscosity of the processed colloidal system [50,51]. In terms of viscosity, the employed pre-homogenized mixture surely resides at the margin of the device's working capacity. The surplus of surfactant which is subjected to the high-pressure homogenization procedure is apparently united in the described shapeless aggregates. Conventional TEM analysis after negative staining confirmed the above observations (Figure 6B). A noteworthy observation regarding the conventional TEM analysis is the fact that a largely intact emulsion structure could be visualized despite the high vacuum and the potential risk of beam damage imminent to this technique. This aspect will be the subject of further investigations.

Summarizing the main conclusions of the microscopic observations, it may be assumed that the highly viscous and dense structure of the macroemulsions is caused by a network of excess surfactant. In case of the nanoemulsion, smaller droplet sizes are created which possess a larger surface area covered by surfactant molecules. Thus, only a small amount of surplus surfactant is left after production which subsequently leads to the observed aggregates which increase in number and size during storage. In case of the macroemulsion, the droplet sizes remain in the micrometer range, thus providing less surface area to be covered by surfactant molecules. Consequently, a larger amount of free surfactant is left which then forms the network-like structure via self-assembly into vesicles or similar structures, possibly approaching a weak liquid crystalline state.

### 3.9. Rheological investigations

The rheological properties of a colloidal system strongly depend on its composition and, more importantly in this case, on the processing conditions. The analysis of these properties is a simple tool to characterize the macroscopic properties of a formulation in an objective way and thus to confirm visual observations [52]. The rheological properties of emulsions are crucial for their application on skin as well as their physical stability. Especially in case of the presented macroemulsions, it may be assumed that the coalescence of oil droplets or other potential destabilization phenomena strongly depend on the rheological properties of the system. These properties are influenced by the oil volume fraction, the droplet size and particle charge as well as by colloidal interactions [53]. In order to obtain an overview about the rheological properties of the different systems, the flow behavior as well as the viscoelastic properties of both blank and drug-loaded systems were analyzed.

**Figure 7.**
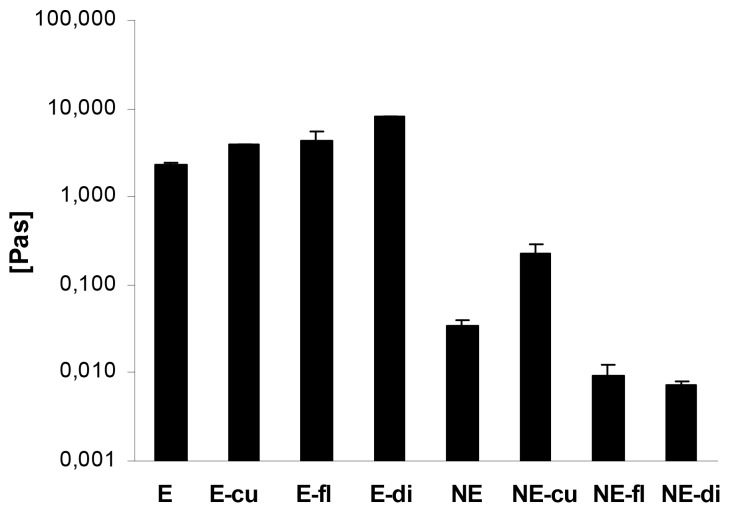
Comparison of the apparent viscosity of macroemulsions (E) and corresponding nanoemulsions (NE) with and without incorporated drug at a shear rate of 15 s^−1^. The effect of the incorporated drugs curcumin (cu), flufenamic acid (fl) and diclofenac acid (di) is demonstrated. Values represent the mean of three formulations (n = 3) and are given ± SD.

In case of the macroemulsions, the flow curves revealed that the dynamic viscosity of the systems decreased with increasing shear rates. This pseudoplastic or shear-thinning flow behavior was found for both blank and drug-loaded macroemulsions. The incorporation of the model drugs led to an increase in viscosity at all shear rates. Figure 7 demonstrates the advantageous effect of drug incorporation on the viscosity of the macroemulsions in an exemplary manner at a shear rate of 15 s^−1^. The apparent viscosity of the macroemulsions at this shear rate was between 2.26 ± 0.10 and 7.92 ± 0.02 Pa·s.

A minor shear-thinning effect was also observed for blank nanoemulsions and nanoemulsions with curcumin. As already indicated, curcumin was partly dispersed in the system, thus changing its overall properties and leading to an increased viscosity and shear-thinning behavior of the nanoemulsion. For all other drug-loaded nanoemulsions, the dynamic viscosity was entirely independent of the applied shear rate which is characteristic for Newtonian flow behavior. Except in the case of curcumin, the incorporation of drugs into the sucrose stearate nanoemulsion led to a decrease of viscosity at all shear rates (Figure 7): nanoemulsions with flufenamic acid and diclofenac acid exhibited an apparent viscosity of 0.01 ± 0.001 Pa·s at all shear rates.

Overall, it was apparent that the viscosity of the macroemulsions was several orders of magnitude higher than that of the nanoemulsions. The apparent viscosity of the blank nanoemulsion was 0.03 ± 0.001 Pa·s at the presented shear rate, while the viscosity of the corresponding blank macroemulsion was 2.26 ± 0.10 Pa·s, which corresponds to a 64-fold increase in viscosity.

An analysis of the flow curves of both macroemulsions and nanoemulsions using the power law model confirmed the above discussed observations. In case of the nanoemulsions, the value of the flow behavior index n was in the order NE diclo > NE fluf > NE blank > NE curc, where especially the values of the nanoemulsions with diclofenac acid and flufenamic acid were close to 1 (n = 0.98 ± 0.03 and n = 0.92 ± 0.08), thus confirming Newtonian flow behavior. In contrast, the flow behavior index n of blank macroemulsions was 0.10 ± 0.01 and was decreased further by incorporation of either drug. A value of n close to zero indicates pronounced shear-thinning behavior of the samples.

Overall, the shear-thinning effect observed for macroemulsions and, to a minor extent, for blank and curcumin-loaded nanoemulsions is consistent with literature. The pseudoplastic nature of the macroemulsions can be explained by the deformation of the emulsion droplets under increasing shear which facilitates their flow. Likewise, nanoemulsions may exhibit shear-thinning behavior due to changes in the droplet shape along the flow channel [22]. In this case, the incorporation of flufenamic acid and diclofenac acid apparently eliminated any such effect by lowering the viscosity of the blank nanoemulsion system, possibly by interacting with surplus amounts of surfactant.

**Figure 8.**
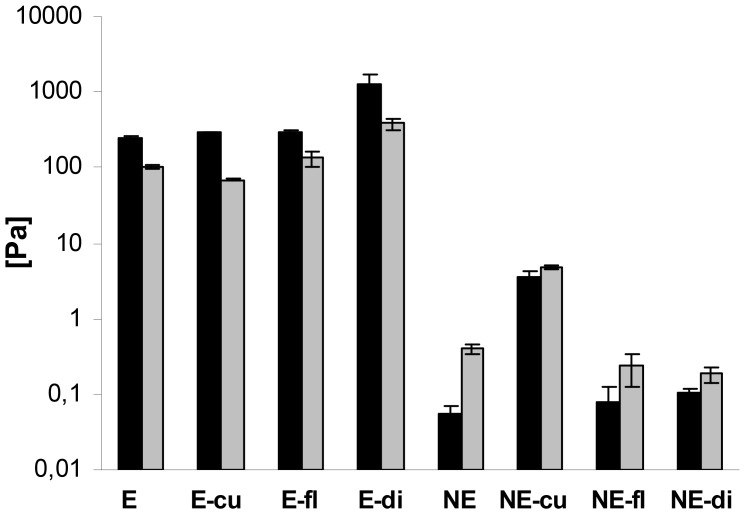
Comparison of the elastic modulus G′ (black bars) and the viscous modulus G″ (grey bars) of macroemulsions (E) and corresponding nanoemulsions (NE) with and without incorporated drug at an oscillatory frequency of 1 Hz. The effect of the incorporated drugs curcumin (cu), flufenamic acid (fl) and diclofenac acid (di) is demonstrated. Values represent the mean of three formulations (n = 3) and are given ± SD.

In addition, oscillatory measurements were performed. In case of the macroemulsions, the elastic modulus G′ was strongly dominating over the viscous modulus G″ at all oscillatory frequencies, which is favorable in terms of physical stability. A comparison of G′ and G″ for all systems at a frequency of 1 Hz is given in Figure 8. The elastic modulus G′ ranged from approximately 250 to 1300 Pa while the viscous modulus G″ ranged from 70 to 380 Pa. Drug incorporation generally increased the values of G′ and G″. The only exception was the curcumin-loaded formulation where G″ was decreased. Overall, both parameters increased at an increasing oscillatory frequency. An increase in the elastic modulus with increasing frequency has been related to the closer packing of microgelled colloidal particles and the resulting larger friction forces between the droplets subjected to shear [54].

In case of the nanoemulsions, the viscous modulus G″ clearly dominated over the elastic modulus G′ (Figure 8). In case of flufenamic acid and diclofenac acid, drug incorporation led to a decrease of the viscous modulus G″, thus confirming their fluidizing effect on the nanoemulsions. In case of curcumin, incorporation led to an increase of the viscous modulus G″ due to undissolved drug. The elastic modulus G′ was increased by drug incorporation in all cases. Again, both parameters G″ and G′ increased with increasing oscillatory frequency. Needless to say, both the elastic and viscous modulus were again several orders of magnitude larger for macroemulsions than for nanoemulsions. While the elastic modulus G′ of blank nanoemulsions was 0.06 ± 0.01 Pa, the corresponding value for blank macroemulsons was 248.01 ± 14.97 Pa. This corresponds to a 4409-fold increase in the formulation's elasticity. Likewise, the viscous modulus G″ of blank macroemulsions was 256 times larger than for blank nanoemulsions (0.40 ± 0.05 *versus* 101.83 ± 3.68 Pa).

Overall, the rheological data confirmed that the employed sucrose stearate mixture led to the formation of entirely different emulsion bulk structures by alteration of the production process. A thorough understanding of the bulk structure of an emulsion is necessary to accurately describe its macroscopic mechanical properties. If the droplets within an emulsion are concentrated enough to be deformed, as is obviously the case for the present systems, the mechanical properties of the system can change from viscous to elastic during production due to increased deformation through osmotic pressure [55]. The application of a small shear strain works against the interfacial tension and causes the packed droplets to deform, thereby creating additional interfacial area and storage energy [55]. Likewise, as indicated by the cryo TEM experiments, the macroemulsion structure appeared to be highly symmetrical in terms of distribution of water and oil phase, which may contribute to increasing viscosity and elasticity [56].

The potential of the sucrose ester mixture to stabilize highly elastic macroemulsions is worthy of further investigation. So far, it may be assumed that the ability of the employed sucrose ester to increase the viscosity of emulsions may be related to its potential to self-assembly and the ability of sucrose to form hydrogen bonds and bind water [17].

### 3.10. In vitro skin permeation: franz-cells

The cumulative permeated drug amounts after 24 hours as well as the corresponding drug fluxes for all three model drugs are given in Table 5.

As can be seen, the corresponding macroemulsions and nanoemulsions performed equally well in this experimental setup, leading to highly similar release profiles in all three cases. No statistically significant differences between the different formulation types were obtained in terms of either cumulative drug amounts or drug fluxes (P > 0.05, respectively). In case of curcumin, the achieved permeation rates were negligible despite the adapted acceptor medium. This can be ascribed to the high lipophilicity of curcumin, which is known to be problematic in terms of formulation development and drug delivery [30,31]. However, the skin permeation experiments were not perpetuated for a longer time span so as not to allow maceration effects to take hold.

The small droplet size of the nanoemulsions is associated with a large surface area, which has frequently been reported to result in an enhanced or accelerated release of incorporated drugs and thus an increased biological effect [51,57,58]. Interestingly, no such effect was observed in the present experiments. Indeed, literature shows that a smaller particle size is not necessarily associated with improved drug delivery [59]. In this case, the skin penetration of the model drugs rather appears to be governed by the employed excipients.

Another interesting aspect in this context is the fact that the skin permeation rates of the drugs were not influenced by the strongly increased viscosity of the macroemulsion systems. The microviscosity of the macroemulsions appeared to be comparable to that of the fluid nanoemulsions despite the significant difference in macroviscosity. This phenomenon has been investigated and confirmed in a recent study dealing with gel-like colloidal dispersions of a comparable sucrose stearate mixture [15]. It may thus be assumed that the loose interconnected hydrophilic network observed for the macroemulsions in our study is of a similar nature despite the presence of additional oil droplets. The release of all three model drugs remained impaired by the complex network-like macroemulsion structure.

### 3.11. In vitro skin penetration: tape stripping

The results of the tape stripping experiments were in good agreement with the results obtained with the franz-cell studies. In summary, neither the particle size nor the viscosity appeared to exert any influence on the penetration behavior of the three model drugs. The penetration depth of the drugs was found to be highly similar for corresponding macroemulsions and nanoemulsions in case of all three model substances (P > 0.05, respectively). The same tendency was observed for the accumulated amounts of drug in the SC as determined by summarizing the drug quantities recovered from the individual tape strips (P > 0.05, respectively). Merely in case of diclofenac acid, slightly if significantly larger drug amounts were recovered from the skin treated with the macroemulsion due to a surplus of drug on the first adhesive film (P < 0.05). Table 6 shows a comparison of the macroemulsions and nanoemulsions in terms of penetrated drug amounts and the penetration depth of the drugs. The latter is expressed both in absolute values (μm) and as percentage of the entire SC thickness, which was found to be 6.59 ± 0.47 μm for the employed porcine ears after removal of the entire SC via tape stripping (n = 4).

**Figure 9.**
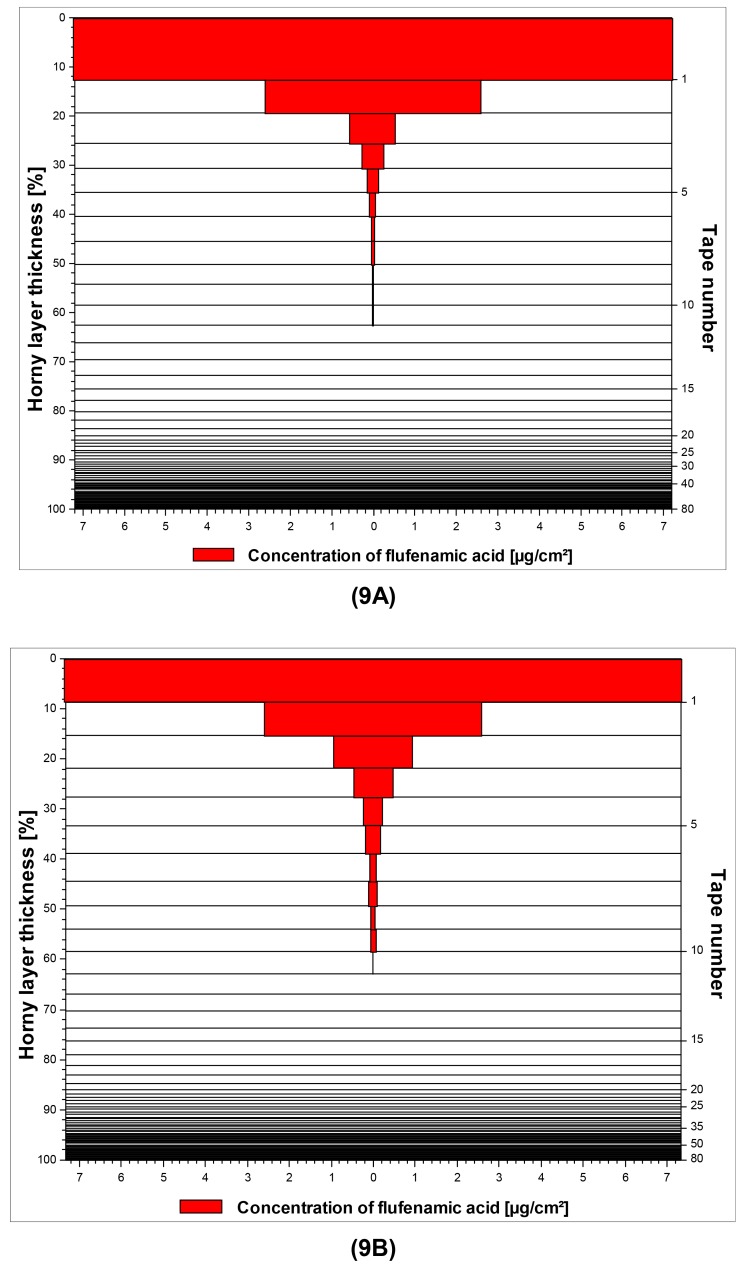
*In vitro* skin penetration profiles of flufenamic acid applied topically in macroemulsions **(9A)** and nanoemulsions **(9B)**. The data were obtained via tape stripping using porcine ear skin (n = 8, respectively).

A graphical illustration of the penetration profiles for flufenamic acid from both macroemulsions and nanoemulsions is given in Figure 9A/9B as a representative example.

The highest skin penetration depth was achieved by by diclofenac acid, which was in good agreement with the results of the franz-cell studies. Concerning curcumin it is interesting to note that unlike the results derived from the franz-cell diffusion studies, satisfying skin penetration was reached in this experimental setup which was comparable to that of flufenamic acid. It may thus be assumed that *in vitro* tape stripping is a more suitable approach to investigate the skin penetration of highly lipophilic drugs such as curcumin. Additional *in vivo* tape stripping studies will be conducted with the curcumin-loaded formulations to confirm these results.

### 3.12. Comparison of in vitro studies: franz-cells vs. tape stripping

Since the classical experimental set-up using franz-type diffusion cells may be influenced by interactions between the receptor medium and the model skin which may affect the barrier properties [60], additional tape stripping experiments were performed to avoid the influence of the receptor medium and to provide a more practically orientated experimental setup with a short finite-dose application.

Under *in vivo* conditions, there is of course a high tissue clearance due to the bloodstream which cannot be simulated with the excised porcine ear. However, the results of our study showed that in the case of curcumin, hardly any skin diffusion was obtained with franz-type diffusion cells over the course of 24 hours despite the presence of a large amount of ethanol in the receptor medium, which offers a high solubilizing capacity for curcumin. In contrast, a penetration profile similar to that of the other drugs was achieved by *in vitro* tape stripping.

As expected, the skin penetration and skin permeation data obtained by the different experimental setups were not entirely comparable in terms of numerical values. In case of curcumin, a reasonable penetration into the stratum corneum was determined by tape stripping (around 50 μg cm^−2^) while no transdermal permeation was observed with franz-type diffusion cells despite the adapted receptor medium. In case of flufenamic acid, the amounts detected in the skin by tape stripping and in the receptor medium after 24 hours were of a similar order of magnitude (around 25 μg cm^−2^, respectively). In case of diclofenac acid, a comparatively smaller amount of drug was obtained by tape stripping (around 25 μg cm^−2^) than was recovered from the receptor medium (around 145 μg cm^−2^). Since it is well-known that diclofenac as an acid is difficult to deliver, it may be assumed that as for curcumin, the results obtained with franz-type diffusion cells are not representative of the *in vivo* situation. In case of curcumin, an underestimation of the skin penetration potential resulted while in case of diclofenac, an overestimation was obtained. The inconsistent experimental results for diclofenac might be related to the different types of setup, the different types of skin employed for the experiments and related differences in pH gradients across the skin [61]. Overall, it may be concluded that tape stripping experiments deliver by far more realistic data than franz-type diffusion cell studies. Comparative *in vivo* tape stripping experiments are envisioned in order to confirm the relevance of the data obtained by *in vitro* tape stripping.

## 4. Conclusions

Sucrose stearate of an intermediate HLB value can be employed to design innovative lipid-based drug delivery systems which possess improved texture for dermal application while at the same time providing unimpeded drug release properties. The exact nature of the fluffy hydrophilic network surrounding the O/W emulsion droplets of various sizes should be subject to further investigations in order to specifically create desired properties of similar new vehicles. In the context of our investigations, additional *in vivo* tape stripping experiments are planned to gain a more realistic estimation of the penetration behavior of the newly developed formulations.

## Figures and Tables

**Table 1.** Basic composition of macroemulsions (E) and nanoemulsions (NE).

**Excipients**	**Emulsion composition (% w/w)** **E = NE**
PCL-liquid	20
Sucrose stearate S-970	5.0
Potassium sorbate	0.1
Model drug	0.5
Distilled water to	100

**Table 2.** Physicochemical properties of fresh **(2A)** and stored **(2B)** blank and drug-loaded macroemulsions (E) after high-shear dispersion with an ultra-turrax. Measurements were performed in triplicate on a Mastersizer 2000 (Malvern, UK) at 25 °C. The samples were diluted with distilled water (1:1000 v/v) and stirred slightly prior to analysis. The parameters shown are the mean particle size expressed as volume weighted mean D [4,3], surface weighted mean D [3,2] and volume median diameter d(0.5) as well as the d(0.1) and d(0.9) values and the span. All values represent the mean of three formulations (n = 3) in μm and are given ± SD.

**(2A) Freshly prepared samples**				

*Parameters*	E blank	E curc	E fluf	E diclo
D [4,3]	06.94 ± 0.97	09.72 ± 1.92	08.56 ± 0.05	11.80 ± 0.42
D [3,2]	03.70 ± 0.15	04.14 ± 0.04	04.46 ± 0.02	05.43 ± 0.36
d (v, 0.1)	01.74 ± 0.04	01.73 ± 0.01	01.72 ± 0.01	02.04 ± 0.16
d (v, 0.5)	05.16 ± 0.26	06.80 ± 0.07	08.26 ± 0.04	11.29 ± 0.45
d (v, 0.9)	12.58 ± 2.25	14.55 ± 0.41	15.44 ± 0.09	22.09 ± 0.42
Span	02.09 ± 0.31	01.89 ± 0.04	01.66 ± 0.01	01.78 ± 0.05

**(2B) After 6 months of storage**				

*Parameters*	E blank	E curc	E fluf	E diclo
D [4,3]	25.81 ± 2.44	26.46 ± 08.27	35.33 ± 2.35	33.03 ± 3.89
D [3,2]	08.71 ± 0.38	06.57 ± 00.72	11.25 ± 0.88	08.79 ± 0.41
d (v, 0.1)	04.18 ± 0.25	02.56 ± 00.25	06.88 ± 0.65	03.41 ± 0.18
d (v, 0.5)	20.04 ± 2.29	13.41 ± 02.57	21.53 ± 1.49	23.54 ± 1.57
d (v, 0.9)	55.31 ± 5.96	49.66 ± 16.30	77.37 ± 3.01	73.61 ± 6.74
Span	02.55 ± 0.05	03.45 ± 00.51	03.28 ± 0.13	02.98 ± 0.08

**Table 3.** Physicochemical properties of blank and drug-loaded nanoemulsions (NE) after 16 cycles of high-pressure homogenization. Measurements were performed in triplicate on a Zetasizer Nano ZS (Malvern, UK) at 25 °C. Samples were diluted with distilled water (1:100 v/v) containing sodium chloride (0.01 mmol) before the experiments to ensure constant conductivity below 0.05 ms/cm. Parameters shown are mean particle size (MPS), zeta potential (ZP), conductivity (cond) and polydispersity index (PDI). Values represent the mean of at least three formulations (n ≥ 3) and are given ± SD.

***Parameters***	**NE blank**	**NE curc**	**NE fluf**	**NE diclo**
Particle size (nm)	116.40 ± 10.22	124.69 ± 2.58	128.33 ± 11.34	114.44 ± 2.42
Zeta potential (mV)	−58.89 ± 14.48	−47.11 ± 1.49	−51.36 ± 2.71	−54.12 ± 4.83
Conductivity (mS/cm)	0.024 ± 0.005	0.017 ± 0.004	0.025 ± 0.005	0.023 ± 0.003
PDI	0.11 ± 0.03	0.25 ± 0.03	0.09 ± 0.01	0.11 ± 0.02

**Table 4.** Physical stability of blank **(4A)** and drug-loaded nanoemulsions (**4B**, shown on systems containing flufenamic acid). Similar results were obtained for all drug-loaded formulations. Experiments were performed in triplicate (n = 3) in regular intervals over an observation period of six months. The indicated parameters are the mean particle size (MPS), polydispersity index (PDI) and zeta potential (ZP). Numbers are given as means ± SD.

**(4A)**			

time (weeks)	**MPS (nm) ± SD**	**NE blank PDI ± SD**	**ZP (mV) ± SD**
0	116.40 ± 10.22	0.11 ± 0.03	−61.73 ± 10.50
4	116.07 ± 09.93	0.12 ± 0.03	−64.13 ± 07.10
8	116.53 ± 08.56	0.13 ± 0.02	−60.91 ± 04.08
12	118.31 ± 08.28	0.12 ± 0.02	−61.27 ± 16.41
16	117.28 ± 08.05	0.13 ± 0.01	−60.70 ± 06.09
20	120.11 ± 08.93	0.13 ± 0.02	−64.69 ± 04.00
24	121.71 ± 09.61	0.15 ± 0.04	−64.76 ± 06.03

**(4B)**			

time (weeks)	**MPS (nm) ± SD**	**NE fluf PDI ± SD**	**ZP (mV) ± SD**
0	128.28 ± 09.61	0.09 ± 0.01	−49.03 ± 00.37
4	128.01 ± 10.01	0.10 ± 0.01	−49.86 ± 02.05
8	125.06 ± 10.06	0.10 ± 0.01	−49.73 ± 04.38
12	126.55 ± 09.16	0.09 ± 0.01	−50.77 ± 04.64
16	129.94 ± 10.47	0.09 ± 0.01	−52.16 ± 05.13
20	132.92 ± 11.46	0.09 ± 0.02	−48.38 ± 02.13
24	129.12 ± 10.78	0.08 ± 0.01	−55.20± 04.40

**Table 5.** Skin permeation rates of curcumin (curc), flufenamic acid (fluf) and diclofenac acid (diclo) from macroemulsions (E) and nanoemulsions (NE) expressed as cumulative permeated drug amounts (μg·cm^−2^) and mean drug fluxes (J, μg·cm^−2^·h^−1^). At least five experiments were performed for each formulation (n ≥ 5); indicated values are means ± SD.

**Formulation**	**Cumulative drug amount after 24h ± SD (μg·cm^−2^)**	**Mean drug flux ± SD (J, μg·cm^−2^·h^−1^)**
E curc	n.d.	n.d.
NE curc	0.71 ± 0.96	0.04 ± 0.06
E fluf	27.27 ± 7.18	1.28 ± 0.34
NE fluf	24.48 ± 5.13	1.15 ± 0.24
E diclo	140.30 ± 27.36	6.66 ± 1.28
NE diclo	147.37 ± 31.05	6.92 ± 1.44

**Table 6.** Skin penetration data of curcumin (curc), flufenamic acid (fluf) and diclofenac acid (diclo) from macroemulsions (E) and nanoemulsions (NE) as determined by tape stripping. At least six experiments were performed for each formulation (n ≥ 6); indicated values are means ± SD.

**Formulation**	**Penetrated drug amount** **[μg/cm^2^]**	**Penetration** **[μm]**	**depth** **[% of SC]**
E curc	45.22 ± 6.61	3.75 ± 0.53	56.79 ± 08.01
NE curc	51.07 ± 4.17	3.21 ± 0.81	48.61 ± 12.24
E fluf	21.93 ± 6.29	3.08 ± 1.29	46.65 ± 19.62
NE fluf	24.02 ± 7.03	3.61 ± 0.95	54.67 ± 14.36
E diclo	27.98 ± 4.43	5.62 ± 0.65	85.22 ± 09.83
NE diclo	20.72 ± 2.58	4.79 ± 0.95	72.70 ± 14.40
